# Expression of Stress-Induced Genes in Bronchoalveolar Lavage Cells and Lung Fibroblasts from Healthy and COPD Subjects

**DOI:** 10.3390/ijms25126600

**Published:** 2024-06-15

**Authors:** Martin Garcia-Ryde, Nicole M. D. van der Burg, Frida Berlin, Gunilla Westergren-Thorsson, Leif Bjermer, Jaro Ankerst, Anna-Karin Larsson-Callerfelt, Cecilia K. Andersson, Ellen Tufvesson

**Affiliations:** 1 Respiratory Medicine, Allergology and Palliative Medicine, Department of Clinical Sciences, Lund, Lund University, 221 84 Lund, Sweden; martin.garcia-ryde@med.lu.se (M.G.-R.); nicole.van_der_burg@med.lu.se (N.M.D.v.d.B.); leif.bjermer@med.lu.se (L.B.); jaro.ankerst@med.lu.se (J.A.); 2 Respiratory Cell Biology, Department of Experimental Medical Science, Lund University, 221 84 Lund, Sweden; frida.berlin@med.lu.se (F.B.); cecilia.andersson@med.lu.se (C.K.A.); 3 Lung Biology, Department of Experimental Medical Science, Lund University, 221 84 Lund, Sweden; gunilla.westergren-thorsson@med.lu.se (G.W.-T.); anna-karin.larsson_callerfelt@med.lu.se (A.-K.L.-C.)

**Keywords:** COPD, cigarette smoke extract, lung fibroblast, bronchoalveolar lavage, gene expression, ER stress, protein

## Abstract

Chronic obstructive pulmonary disease (COPD) is commonly caused from smoking cigarettes that induce biological stress responses. Previously we found disorganized endoplasmic reticulum (ER) in fibroblasts from COPD with different responses to chemical stressors compared to healthy subjects. Here, we aimed to investigate differences in stress-related gene expressions within lung cells from COPD and healthy subjects. Bronchoalveolar lavage (BAL) cells were collected from seven COPD and 35 healthy subjects. Lung fibroblasts were derived from 19 COPD and 24 healthy subjects and exposed to cigarette smoke extract (CSE). Gene and protein expression and cell proliferation were investigated. Compared to healthy subjects, we found lower gene expression of CHOP in lung fibroblasts from COPD subjects. Exposure to CSE caused inhibition of lung fibroblast proliferation in both groups, though the changes in ER stress-related gene expressions (ATF6, IRE1, PERK, ATF4, CHOP, BCL2L1) and genes relating to proteasomal subunits mostly occurred in healthy lung fibroblasts. No differences were found in BAL cells. In this study, we have found that lung fibroblasts from COPD subjects have an atypical ER stress gene response to CSE, particularly in genes related to apoptosis. This difference in response to CSE may be a contributing factor to COPD progression.

## 1. Introduction

Chronic obstructive pulmonary disease (COPD), characterized by emphysema and bronchitis, is primarily caused by cigarette smoke or other air pollutants. These airway triggers give rise to a stress response in the cells of the lungs [1] that involves different mechanisms by several pathways to manage the components of exposure. The response in healthy compared to COPD airways during exposure can differ significantly, though further investigation is required to determine the pathways involved in this difference.

In a previous study, we showed that the endoplasmic reticulum (ER) was more disorganized in lung fibroblasts from subjects with COPD compared to healthy never-smokers and healthy ever-smokers [2]. When exposed to chemical stressors, we found an altered response in ER stress genes in the lung fibroblasts from COPD subjects compared to healthy subjects. Even though cigarette smoke plays a prevalent role in COPD development, it is not known how cigarette smoke affects the ER stress response in lung fibroblasts. Other studies have shown that cigarette smoke can induce oxidative stress [3,4,5] and lung fibroblast differentiation through ER stress induction [6]. Oxidative stress leads to the activation of oxidation resistance 1 (OXR1), activating genes related to oxidative stress protection. Oxidative stress can also cause protein misfolding and accumulation of proteins [7]. Normally, the proteasome is responsible for breaking down proteins since the accumulation of misfolded proteins can also lead to ER stress and the unfolded protein response (UPR). The proteasome is composed of several subunits, such as proteasome 20S subunit alpha 1 (PSMA1), proteasome 20S subunit beta 6 (PSMB6), and proteasome 26S subunit non-ATPase 11 (PSMD11). If this fails, the UPR leads to alterations in a multitude of genes to either correct the unfolded/misfolded proteins or to terminate the cell [8].

The UPR is activated by three ER membrane-associated proteins: activating transcription factor-6 (ATF6) that is translocated and cleaved to become active in regulating the UPR altered genes; inositol-requiring enzyme 1 (IRE1) that cleaves X-box-binding protein 1 that then becomes a strong transcription factor; and lastly, the protein kinase RNA-like endoplasmic reticulum kinase (PERK) that phosphorylates eucaryotic initiation factor 2α (eIF2α) and nuclear erythroid 2-related factor 2 (NRF2) [9]. Downstream of this activation of the UPR, eIF2α initiates activating transcription factor 4 (ATF4). ATF4 and NRF2 alter the expression of UPR-targeted genes. If the UPR cannot solve the problems with the unfolded/misfolded proteins, PERK can activate C/EBP homologous protein transcription factor (CHOP) that can, together with the IRE1-activated c-Jun N-terminal kinase (JNK), lead to apoptosis. The cell could avoid apoptosis by activating the apoptosis regulator Bcl-2 (Bcl-2). Despite the importance of the UPR and its association to smoking, little is known about smoke- and pollutant-induced alterations of stress-related genes in the airways of COPD patients. Our hypothesis is that the stress response is deficient in cells from subjects with COPD and, thereby, is a contributing factor to disease progression.

The aim of the present study was, therefore, to assess the expression of stress-related genes in bronchoalveolar lavage (BAL) cells and in lung fibroblasts from COPD subjects compared to healthy subjects at baseline. We also investigated if the stress-related pathways were affected differently in lung fibroblasts from healthy and COPD subjects by cigarette smoke extract (CSE).

## 2. Results

### 2.1. Expression of Stress-Related Genes in BAL Cells

**Figure 1:**
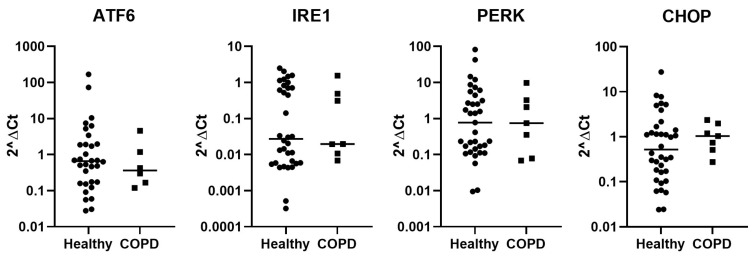
Gene expression of stress-related genes (ATF6, IRE1, PERK, and CHOP) in BAL cells from healthy (n = 35) and COPD (n = 7) subjects. Data are presented as individual values and median.

The difference in expression levels of stress-related genes among total BAL cells was compared between healthy and COPD subjects. There was no significant difference in the expression of the four genes relating to ER stress (ATF6, IRE1, PERK, or CHOP) (Figure 1). Other stress-related genes (PSMB6, PSMA1, PSMD11, OXR1, Bcl-2, and Nrf2) were investigated but no significant difference was found (Figure S1). No differences were found when investigating only the current smoking COPD subjects compared to current smoking healthy subjects.

The proportions of the different cell types (macrophages, neutrophils, eosinophils, and lymphocytes) did not differ significantly between healthy and COPD subjects (Supplemental Table S4). An adjustment of the gene expression relative to the proportion of each cell type present in the BAL (by multiplying the level of the stress-related genes by the proportion of the respective BAL cell type) was performed, but also found no differences between healthy and COPD subjects.

**Figure 2:**
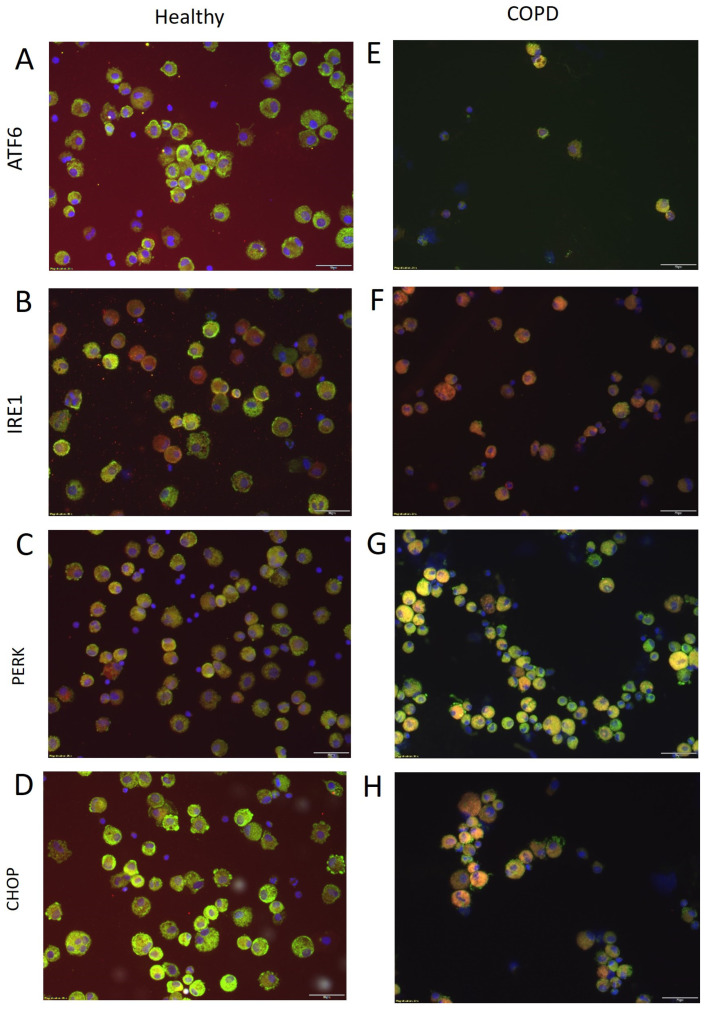
Representative images of immunofluorescence stainings of BAL cells from healthy subjects (**A**–**D**) and COPD patients (**E**–**H**). Red represents the different ER stress mediators (ATF6, IRE1, PERK, and CHOP, respectively), green represents the ER (staining of protein disulfide isomerase), and blue represents the nuclei (DAPI). Yellow indicates co-localization of ER and the ER stress mediator. Scale bar equals 20 μm.

Immunofluorescence stainings of ER together with ER stress mediators in BAL cells were performed to visualize the expression of ATF6, IRE1, PERK, and CHOP in healthy (Figure 2A–D) and COPD subjects (Figure 2E–H). Co-staining of the ER and the ER stress mediators was observed in macrophage, eosinophil, and neutrophil cells, but was not present in lymphocytes (identified by morphology).

### 2.2. Expression of Stress-Related Genes in Lung Fibroblasts

Immunofluorescent staining confirmed a disorganized ER in lung fibroblasts from subjects with COPD compared to healthy subjects (Figure S2), suggesting ER stress, in accordance with previous findings [2].

**Figure 3:**
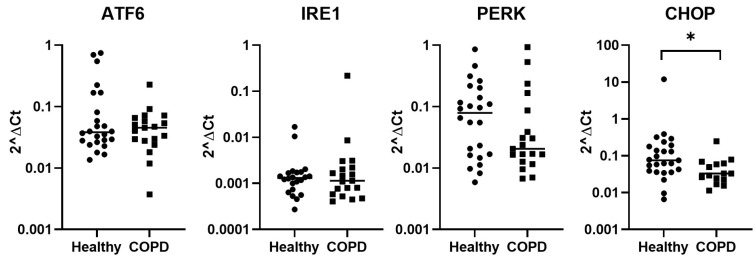
Baseline gene expression of stress-related genes in lung fibroblasts from healthy and COPD subjects. In ATF6, IRE1, and PERK: healthy n = 27 and COPD n = 22, and in CHOP: healthy n = 25 and COPD n = 16. Data are presented as individual values and median. The Mann–Whitney test was used for statistical analysis. * = *p* < 0.05.

Baseline gene expression of stress-related genes in lung fibroblasts was investigated. There were significantly lower levels of CHOP in COPD compared to healthy subjects (*p* = 0.015, Figure 3). ATF6, PERK, and IRE1 gene expression did not show any difference between COPD and healthy subjects. Additional stress-related genes (PSMB6, PSMA1, PSMD11, OXR1, Bcl-2, and Nrf2) were also investigated but no significant difference could be found between healthy and COPD (Figure S3).

A subgrouping based on smoking status was done to highlight the effects of smoking (Figure S4). Despite the low number of individuals, a significantly lower level of CHOP expression was seen in lung fibroblasts from ex- and current smokers in COPD subjects when compared specifically to the lung fibroblasts from healthy never-smokers (*p* = 0.012 and *p* = 0.042, respectively). In addition, IRE1 was found to be upregulated in current smokers with COPD compared to ex-smokers with COPD (*p* = 0.014), but no significant difference was observed when compared to lung fibroblasts from healthy subjects.

The expression of ER stress-related genes, at baseline, was also investigated according to if the lung fibroblasts were derived from distal compared to central airways of healthy and COPD subjects (Figure S5). Healthy subjects had higher levels of CHOP in lung fibroblasts from central compared to distal airways (*p* = 0.010). The overall lower level of CHOP in COPD subjects was, thus, due to lower levels of CHOP in lung fibroblasts from central airways in COPD subjects compared to the central airways in healthy subjects (*p* = 0.0022). Similarly, there was a tendency (*p* = 0.095) of lower levels of PERK in central airways in COPD compared to healthy subjects. In addition, there was a lower expression of PSMB6 in the central airway fibroblasts compared to distally derived from both healthy (*p* = 0.0160) and COPD subjects (*p* = 0.029).

**Figure 4:**
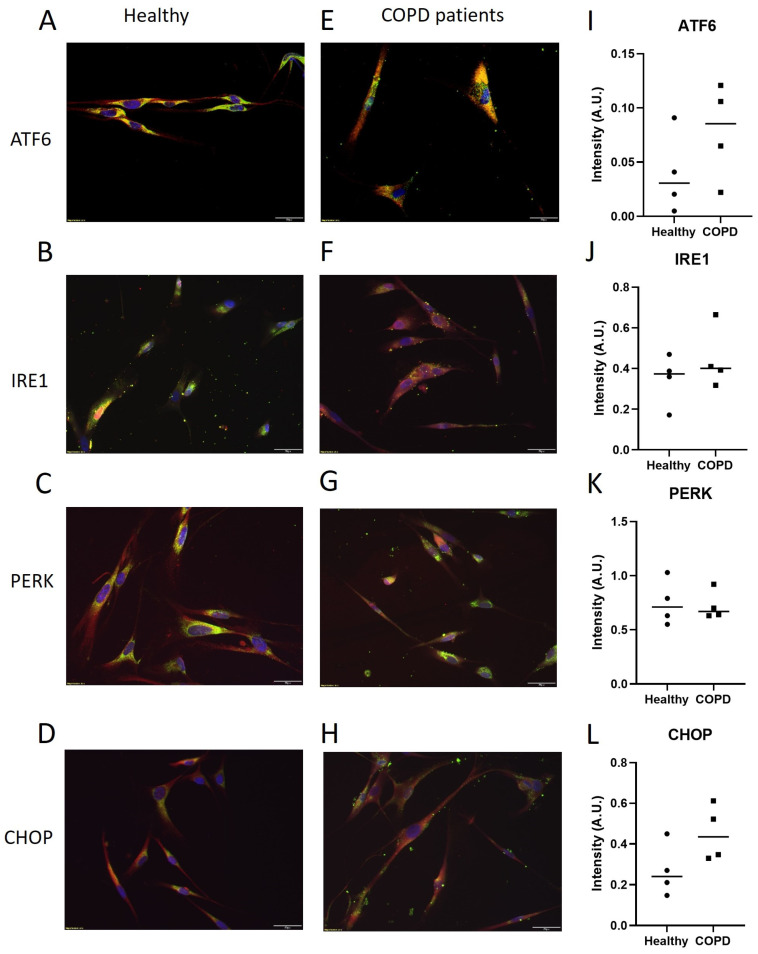
Lung fibroblasts from healthy smokers (**A**–**D**) and COPD patients (**E**–**H**), cultured from distal lung tissue derived from resections. Representative images of immunofluorescence stainings (n = 4 in each group) with red representing the different ER stress mediators (ATF6, IRE1, PERK, and CHOP, respectively), green representing the ER (staining of protein disulfide isomerase), and blue representing the nuclei (DAPI). Yellow indicates co-localization of ER and the respective ER stress mediator. Intensity measurement of Western blot (**I**–**L**) of the respective ER stress mediators, presented as individual dots and median. Scale bar equals 20 μm.

Staining of the ER stress mediators in lung fibroblasts was performed to visualize the localization of ATF6, IRE1, PERK, and CHOP in healthy (Figure 4A–D) and COPD subjects (Figure 4E–H). The stainings showed co-localization of the ER and the ER stress mediators in the lung fibroblasts. There were no quantifiable differences in the Western blot of the proteins between the two groups (Figure 4I–L), though, both ATF6 and CHOP tended to be higher in lung fibroblasts from COPD subjects compared to healthy subjects.

### 2.3. The Effect of CSE on Cell Proliferation

**Figure 5:**
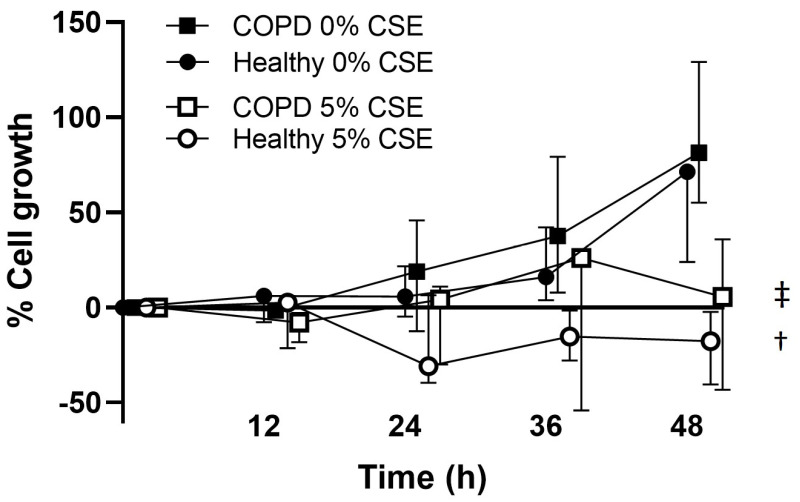
Lung fibroblast proliferation during 48 h measured by HoloMonitor. The graph presents cell growth as % of time point 0. Lung fibroblasts from healthy subjects (n = 3) and COPD patients (n = 3) were investigated without (0% CSE) or with Cigarette smoke extract (5% CSE). † and ‡ = significantly different trend (*p* = 0.05) from 0 h to 48 h between 0 and 5% CSE within healthy and COPD subjects, respectively. Friedman’s test was used for statistical analysis of differences in trends over time between 0 and 5% CSE.

To investigate the potential effect of CSE on lung fibroblasts, the proliferation of lung fibroblasts from healthy and COPD subjects was quantified using the HoloMonitor. We found that the proliferation of lung fibroblasts from both healthy and COPD subjects was inhibited during exposure with 5% CSE at 48 h (*p* = 0.05 in both, Figure 5), with no significant differences between COPD and healthy subjects.

### 2.4. Protein Expression of ER Stress Mediators after CSE Exposure

**Figure 6:**
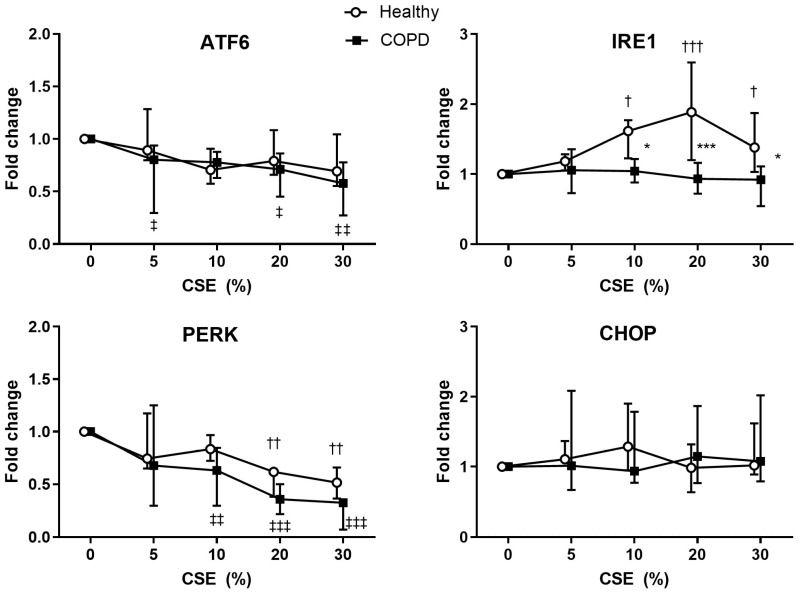
Protein expressions of ER stress mediators ATF6, IRE1, PERK, and CHOP in lung fibroblasts from healthy and COPD subjects analyzed by Western blot. The fibroblasts were stimulated with CSE (0–30%) for 8 h and normalized against the housekeeping protein GAPDH. The values are presented as relative to the protein with unstimulated cells (0% CSE) of the respective individual, n = 4 for ATF6, IRE1, and CHOP, and n = 3 for PERK. Data are presented as median values and interquartile range. Mixed effects analysis was used for statistical analysis. * = significant difference between healthy and COPD subjects at the respective % CSE. † and ‡ = significant difference between 0% CSE and the respective % CSE within healthy and COPD subjects, respectively. One symbol = *p* < 0.05, two symbols = *p* < 0.01 and three symbols = *p* < 0.001.

Since cell proliferation was inhibited by CSE at 48 h (Figure 5), we investigated the protein levels of the different stress pathways (ATF6, IRE1, PERK, and CHOP) at a preceding timepoint (8 h) after exposure to different concentrations of CSE in lung fibroblasts from healthy and COPD subjects (Figure 6). ATF6 and PERK was decreased in response to increasing concentrations of CSE, more so in lung fibroblasts from COPD subjects (ATF6: CSE 5% *p* = 0.030, 10% *p* = 0.099, 20% *p* = 0.028, 30% *p* = 0.0031, and PERK: CSE 5% *p* = 0.074, 10% *p* = 0.0079, 20% *p* = 0.0002, 30% *p* < 0.0001 compared to 0%), but also in lung fibroblasts from healthy subjects (ATF6: CSE 10% *p* = 0.063 and 30% *p* = 0.10, and PERK: CSE 20% *p* = 0.0036, 30% *p* = 0.0024 compared to 0%). In contrast, the levels of IRE1 were increased in response to CSE in lung fibroblasts from healthy subjects (CSE 10% *p* = 0.013, 20% *p* = 0.0002, 30% *p* = 0.043 compared to 0%), but not in lung fibroblasts from COPD subjects.

There was, subsequently, a difference between healthy and COPD subjects’ lung fibroblasts in the IRE1 response (CSE 10%: *p* = 0.049, 20% *p* = 0.0004, and 30% *p* = 0.023 compared to 0%), but not in ATF6, PERK, or CHOP.

### 2.5. Expression of ER Stress-Related Genes in CSE Stimulated Lung Fibroblasts

**Figure 7:**
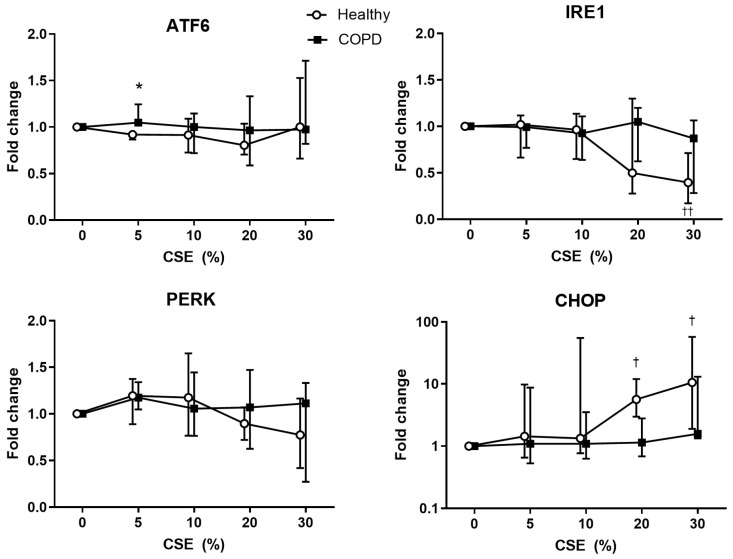
Gene expression of stress-related genes, ATF6, IRE1, PERK, and CHOP, in lung fibroblasts from healthy (n = 9) and COPD (n = 7) after stimulation with 0–30% cigarette smoke extract (CSE) for 4 h. Data are presented as median and interquartile range. All values are presented as ratios relative to the respective individuals’ result at 0% CSE. Mixed-effects analysis was used for statistical analysis. * = *p* < 0.05 between healthy and COPD at 5% CSE, † = *p* < 0.05 or †† = *p* < 0.01 significant difference between 0% and the respective concentrations of CSE in healthy subjects.

At an earlier timepoint (4 h), we investigated the gene expression response to increasing concentrations of CSE stimulation in lung fibroblasts from healthy and COPD subjects (Figure 7). Though the ATF6 protein expression decreased in both, more so in the COPD, the AFT6 gene expression was unchanged from baseline in both. Healthy subjects, though, had significantly less gene expression at 5% CSE exposure (*p* = 0.031) than the corresponding COPD.

PERK protein expression was similar to ATF6, though the healthy lung fibroblasts tended to have decreased gene expression only at the higher CSE concentrations. The higher protein expression in IRE1 at 8 h was preceded by significantly lower gene expression at 4 h in healthy lung fibroblasts (CSE 20% *p* = 0.086 and 30% *p* = 0.0035 compared to 0%). In contrast, despite the unchanged protein expression of CHOP in both groups, healthy subjects had a significantly increased CHOP gene expression response to higher concentrations of CSE (CSE 20% *p* = 0.12, and 30% CSE: *p* = 0.092 compared to 0%). Additional stress-related genes (Bcl2, Nrf2, PSMA1, and OXR1) were investigated but no significant difference between COPD and healthy subjects could be found (Figure S6).

**Figure 8:**
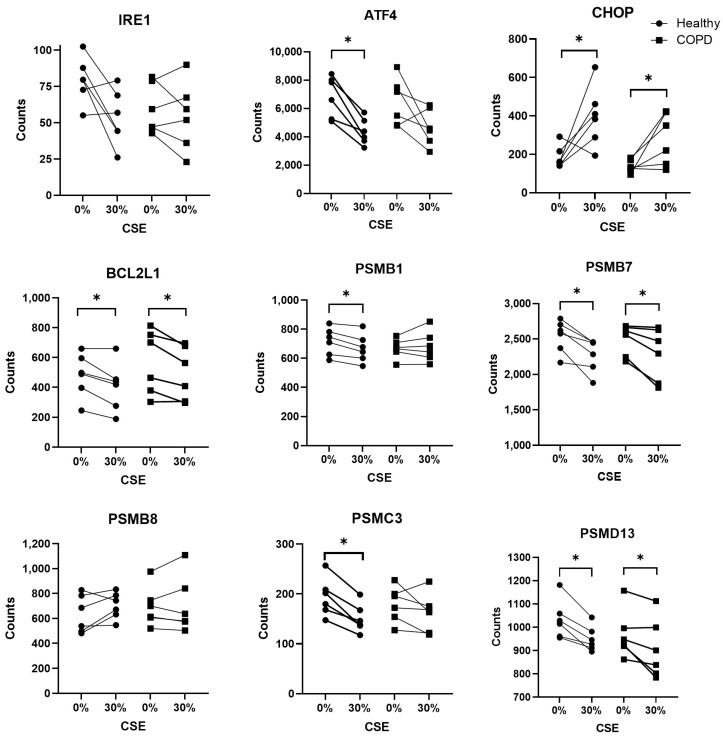
Gene expression of stress-related genes in lung fibroblasts from healthy (●; n = 6) and COPD (**▪**; n = 6) after stimulation with 0 or 30% CSE for 4 h and analyzed using NanoString. The data are presented as the median and the interquartile range. The Wilcoxon signed-rank test was used for the analysis of the samples. * = *p* < 0.05.

Since there was a tendency for differential expression in both IRE1 and PERK at 30% CSE, additional ER stress-related genes were further investigated (Figure 8) from a previously reported and broadly assessed NanoString panel [10]. ATF4, a component downstream of PERK, was decreased in healthy subjects after 30% CSE exposure (*p* = 0.028). Expression of CHOP was increased in healthy subjects (*p* = 0.046) (similarly to the qPCR results presented in Figure 7) and in COPD subjects (*p* = 0.046) after 30% CSE exposure. The expression of Bcl2L1 was decreased in both healthy (*p* = 0.046) and COPD subjects (*p* = 0.046) in response to 30% CSE. Unlike the qPCR, the more sensitive NanoString also found changes in the gene expression of several proteasomal units. This included decreased expression of PSMB1 and PSMC3 in healthy subjects (*p* = 0.028 and *p* = 0.028, respectively), and decreased expression of PSMB7 and PSMD13 in both healthy (*p* = 0.028 and *p* = 0.028, respectively) and COPD subjects (*p* = 0.028 and *p* = 0.046, respectively) after 30% CSE stimulation. Overall, there were more changes in gene expression in the healthy lung fibroblasts than the COPD lung fibroblasts when exposed to CSE.

## 3. Discussion

This study investigated the expression of stress-related genes in BAL cells and lung fibroblasts from healthy and COPD subjects. BAL cells did not present with any clear differences in ER stress expression that might be due to their mixed cell types. Lung fibroblasts, however, displayed multiple differences in ER stress expression between healthy and COPD subjects with and without CSE exposure. At baseline, COPD lung fibroblasts may be less prone to apoptosis (based on CHOP expression) than healthy ones, particularly non-smoking healthy. After CSE exposure, there was a mixed ER stress response whereby healthy lung fibroblasts tended to incur more changes (relative to baseline) than COPD lung fibroblasts. Moreover, there was a general reduction in the proteosome response related to repairing misfolded proteins primarily in healthy lung fibroblasts.

The intention of using BAL cells, containing a mixture of cell types, was to investigate the total cell population in the airway lumen to explore the total ER stress response. It is not known, however, how stress-related genes are expressed and if they are differently expressed in these different cell types. We adjusted the expression of the different ER stress response genes to the respective cell type proportions, but still no differences were found between healthy and COPD subjects. Nonetheless, we believe future investigations of ER stress responses within a BAL cell type would however result in different levels of response between COPD and healthy cells.

The lower expression of CHOP in lung fibroblasts from COPD subjects could indicate that the cells are less prone to apoptosis. Similar findings were reported by Korfer M. et al., investigating the expression of CHOP in COPD lung tissue homogenate compared to healthy and IPF samples [11]. The lower expression levels of CHOP that we found in COPD were specifically different compared to lung fibroblasts from non-smoking healthy subjects. Other studies have shown that small airway fibroblasts from patients with COPD are senescent and associated with fibrotic properties [12]. It is interesting to note, therefore, that the baseline status of ER stress is different in lung fibroblasts from COPD compared to healthy subjects, and most probably associated with smoking status.

Bronchial epithelial cells from patients with severe emphysema have been shown to have significantly higher levels of CHOP [13]. This increase may be due to the hypoxic stress in severe emphysema that has been shown to upregulate CHOP in mouse lung epithelial cells [14]. This supports our findings related to higher gene expression levels of CHOP in lung fibroblasts derived from the central airways of the healthy subjects only. A discrepancy between airway and parenchymal lung fibroblasts has also previously been shown in subjects with COPD in the context of extracellular matrix composition [15]. In addition, the level of CHOP is predominantly higher in never-smoker healthy subjects who are in the group of centrally derived fibroblasts. These factors might contribute to the different directions of mRNA and protein levels of CHOP in healthy versus COPD subjects since the protein levels (Figure 4) were in lung fibroblasts from distally derived tissue of smoking subjects.

To support our hypothesis that lung fibroblasts respond to CSE by inducing apoptosis, both groups increased pro-apoptotic CHOP and downregulated anti-apoptotic BCL2 after CSE exposure. Interestingly, only healthy lung fibroblasts consistently decreased ATF4, a stimulant of CHOP [16], indicating some negative feedback and control over the apoptosis signaling that was not reflected in the COPD cells.

After exposure to CSE, the healthy lung fibroblasts made several more changes than the COPD to the ER stress pathway [10]. Healthy lung fibroblasts seem to follow the traditional ER stress pathway to apoptosis by decreasing several of the proteasomal units that try to repair the misfolding, reducing the attempt to save the cell. This result was similar to the CSE-stimulated J774 macrophages [17]. The response from COPD lung fibroblasts was significantly less consistent. This inconsistent response could help explain why inhibiting parts of the ER stress pathway in COPD was concluded to be complex in a recent review, as an impaired production of ER molecules involved in folding could further induce misfolding of proteins [18].

Different doses and timings were used in the CSE exposure experiments depending on the readout. CSE doses were based on initial experiments where a dose–response analysis of 0–30% CSE using lactate dehydrogenase showed the most apparent response at 20–30% CSE [10]. Proliferation was read at a low dose over a long time since it relied on cell cycle timing. To ensure time for protein and gene expressions, proteins were read at 8 h based on the latest report of protein production times [19] and genes were read at 4 h when the IRE1α pathway is reported to be active post-exposure [20]. In comparison to other CSE exposure studies with human fibroblasts, we used a much higher concentration of CSE than the reported 1.25–5% [21], 2.5–10% [22], 5% [23], or 10% [24]; however, our exposure time was much shorter than these studies that exposed cells to CSE extract during 3 days, 14 days, 3 days, or 1–3 days, respectively. Therefore, we believe that we adjusted the timing and concentration of CSE appropriately to investigate changes in gene expression, protein expression, and cell proliferation before the cell numbers were significantly compromised.

A limitation of this study is that many of the healthy and COPD lung fibroblasts were derived from lobectomies of lung tumor patients and so are from an environment that is different from normal physiological conditions. Even though they are derived at a clinically significant distance from the tumor tissue, we cannot say for certain that they were not changed by the environment that the tumor cells had made or that they were not affected by a systemic pathological status. Another limitation is the unexplored effect of taking cells from a cigarette smoke-polluted environment and culturing them in a clean media environment prior to CSE exposure. Therefore, these findings rely on the memory of response to cigarette smoke only. Lastly, these results are greatly dependent on the time and concentration of CSE exposure; for example, gene expressions are best assessed early and so a higher CSE concentration and short exposure time was applied.

## 4. Material and Methods

### 4.1. Subject Characteristics

A total of 42 subjects (35 healthy and 7 COPD subjects) underwent bronchoscopy where bronchoalveolar lavage (BAL) was collected (Table 1). The median age and gender distribution were similar for the healthy and COPD subjects. Most subjects with COPD were in the GOLD stage 2 of their disease. Most healthy subjects were either never-smokers or current smokers, while the COPD subjects were either current or ex-smokers.

Lung fibroblasts were derived from lung tissue from a total of 43 subjects (24 healthy and 19 COPD subjects) (Table 1). Out of these, lung fibroblasts were derived from biopsies taken in 14 healthy and 10 COPD subjects, and from lung resection tissue derived from lobectomies of lung tumor patients from 10 healthy and 9 COPD subjects. Only eight subjects (all healthy) from whom biopsies were collected were also among the subjects from whom BAL was collected. The median age was similar for the healthy and the COPD subjects. The majority were ex-smokers and among the subjects with COPD there were no never-smokers. Most COPD subjects were in GOLD stage 2, but all four stages were included.

CSE exposure was performed on lung fibroblasts from a subgroup of 16 subjects (9 healthy and 7 COPD subjects, all derived from distally derived resected lung tissue, see Table S1). Most of the subjects were ex-smokers and there were no never-smokers included. The median age was similar for the healthy and COPD subjects. Most of the COPD subjects were in the GOLD 2 stage.

All subjects signed written informed consent, and the study was approved by the Regional Ethical Review board in Lund, Diary numbers 2008/431 and 2015/891, and was performed in accordance with the Declaration of Helsinki.

### 4.2. Bronchoalveolar Lavage

The middle lobe was instilled with 2 × 50 mL of phosphate-buffered saline (PBS), with a recovery of 30–60%, and the BAL was filtered through a 60 μm filter. The fluid was centrifuged at 400 g for 10 min at 4 °C. The supernatant was removed, and the pellet was resuspended and washed once in PBS. After an additional centrifugation, the supernatant was discarded, and a fraction of the cells were subjected to cytospin centrifugation to be used for fluorescence stainings. The remaining cells were lysed in RLT buffer (RNeasy lysis buffer, cat# 79216, Qiagen, Hilden, Garmany,) for analysis of RNA and stored at −80 °C.

### 4.3. Cell Culture of Fibroblasts

During bronchoscopy, central biopsies were taken in the subcarina of the lung. Peripheral primary lung fibroblasts were obtained from parenchyma in lung tissue resected during lobectomies or lung transplantations. Lung fibroblasts were derived from biopsies or resected tissue through growth in DMEM (Sigma-Aldrich art nr D5671, Irvine, UK) supplemented with 10% fetal calf serum (Fischer Scientific art nr 1057-0083, Waltham, MA, USA), 1% *L*-Glutamine (Life Technologies art nr 25030081, São Paulo, Brazil), 1% amphotericin B (Sigma-Aldrich art nr A2942, Israel), and 0.5% gentamicin (Sigma-Aldrich art nr G1272, Jerusalem, Israel) as described previously [25].

### 4.4. Cigarette Smoke Extract Preparation

A modified version of the protocol presented by Kode et al. 2008 [4] was used to make the cigarette smoke extract (CSE). A vacuum was used to smoke research cigarettes from University of Kentucky (purchasing info: Center for Tobacco Reference Products, ref:3R4F, University of Kentucky). One cigarette was smoked for about 3 min per 5 mL of DMEM. The extract was filtered through a 0.45 μm filter, 1% amphotericin B (Sigma-Aldrich art nr A2942, Israel) and 0.5% gentamicin (Sigma-Aldrich art nr G1272, Israel) were added, and it was stored in small aliquots at −80 °C. All experiments were carried out in two batches, equally distributed between experiments on fibroblasts from healthy and COPD subjects.

### 4.5. Cigarette Smoke Extract Exposure

Fibroblasts were grown to 80–90% confluency for CSE exposure experiments. Culture media were complemented with CSE ranging from 0–30% and the cells were harvested after 4 h for RNA analysis using RLT buffer and stored at −80 °C. In parallel, the cell layer was harvested after 8 h in Cell Lysis Buffer II (FNN0021, Invitrogen, Carlsbad, CA, USA) for protein analyses using Western blot. A dose–response analysis using lactate dehydrogenase of 0–30% CSE has previously been presented [10].

### 4.6. RNA Purification and qPCR

The RNA was purified with Qiagen RNeasy Mini Kit (Ref 74104, Hilden, Germany). Then, cDNA synthesis and real time quantitative PCR (RT-qPCR) was performed, as described in Tufvesson E. et al. 2011 [26], and presented as 2^^Δ*Ct*^ where Δ*C_t_* = the *C_t_* value for the housekeeping gene—*C_t_* values for the gene of interest. Primers used are outlined in Supplemental Table S2.

### 4.7. Immunofluorescent Staining

BAL cells cytospun on slides and lung fibroblasts cultured (20,000 cells/well) on glass slides (LabTek Chamber Slide w/Cover Glass Slide Sterile, 4-well, 177399PK, Thermo Scientific Nunc. Thermo Fisher Scientific, 75 Panorama Creek Drive, Rochester, NY, USA) were fixed for 30 min in 4% paraformaldehyde. After washing, the cells were permeabilized in 1% Tween20 in PBS for 5 min at room temperature. The cells were then blocked for 60 min at RT using 2% Normal Goat Serum in PBS, and thereafter incubated with primary antibodies (Supplemental Table S3) diluted in blocking buffer for 60 min. After being washed for 3 × 5 min in PBS, the cells were incubated for 60 min with fluorescence-labelled secondary antibodies diluted in blocking buffer. Secondary antibodies were AlexaFluor488 goat-anti-mouse (conc. 1:600, Life Technologies, A11001) and AlexaFluor555 goat-anti-rabbit (conc. 1:600, Life Technologies, A27039). After another washing step with PBS for 3 × 5 min, mounting media with DAPI (Life Technologies, P36935) was added to the cells to stain the nuclei. A Nikon Eclipse 80i microscope (Tokyo, Japan) with an Olympus DP80 camera and a Nikon Plan Apo 20×/0.95 objective was used for the visualization of the cells. CellSens Dimensions (Olympus, Tokyo, Japan) was used to obtain images.

### 4.8. Western Blot

The protein levels of ER stress mediators after CSE exposure were analyzed using Western blot. Total protein levels were measured using Pierce BCA Protein Assay Kit (Thermo Scientific, 23225), and the same amount of protein sample was mixed with 2× Laemmli loading buffer (Sigma Aldrich, S3491). The samples were heated at 95 °C for 5 min and run on a Mini-PROTEAN TGX stain-free gel (Bio-Rad, 456-8096, Hercules, CA, USA) at 150 V for 1 h in running buffer (Bio-Rad, 161-0772) and thereafter transferred onto a membrane using the Trans-Blot Turbo Transfer pack (Bio-Rad, 1704158) at 25 V for 7 min. The membranes were blocked with 5% milk for 1 h, and then incubated overnight with the primary antibodies (Supplemental Table S3) in 4 °C, and thereafter with the secondary antibodies for 1 h. The secondary antibodies were HRP-conjugated goat-anti-rabbit (conc. 1:2000, Abcam, ab6702, Cambridge, UK) and HRP-conjugated goat-anti-mouse (conc. 1:2000, Life Technologies, A28177, USA). The membrane was developed using Clarity Western ECL substrate (Bio-Rad, 170-5061). Restore PLUS Western Blot Stripping Buffer (Thermo Scientific, 45430) was used according to manufacturer’s instructions to strip the membranes between the separate primary antibodies.

### 4.9. Analyses of Cell Proliferation Using HoloMonitor

Human lung fibroblasts were grown in 6-well plates (Sarstedt TC, Ref. 83,3920, Nümbrecht, Germany) for 24 h prior to CSE exposure and were seeded at a concentration of 10,000 cells/cm^2^. These cells were exposed to either 0% or 5% CSE and studied using the HoloMonitor M4 live cell imaging system (Phase Holographic Imaging, Lund, Sweden). For each sample, 4–7 focus points were set per well and exposure, and images were captured over a time period of 48-h. The average percentage of cell growth relative to the starting number of cells at the 0 h time point was calculated per individual.

### 4.10. NanoString Gene Expression Analysis

A post hoc, in-depth analysis of ER stress and UPR genes was included here from the NanoString analysis of CSE-stimulated gene expressions in lung fibroblasts from experiments completed in Ryde M. et al. 2023 [10]. The experiment used the NanoString nCounter© Human fibrosis V2 Panel (Cat# XT-CSO-HFIB2-12) with 760 genes and the data were analyzed using the supplied nSolver 4.0 software. The lower detection limit was set to 23 counts based on negative controls. The experiment assessed an accelerated exposure protocol of 30% CSE for 4 h to analyze lung fibroblast gene expression.

### 4.11. Statistics

Data are presented as median and interquartile range. IBM SPSS Statistics 27 was used for statistical analysis and GraphPad Prism 9 was used for graphical presentations. The Mann–Whitney U test was used to compare results between two groups (healthy versus COPD subjects), while the Kruskall–Wallis test was used for analyzing results from BAL cells and lung fibroblasts when comparing more than two groups (between smoking statuses). Mixed-effects analysis or repeated measures 2-way ANOVA was used for paired samples, with the uncorrected Dunn’s test or uncorrected Fisher’s LSD, respectively, for analysis of the CSE-exposed fibroblasts at the different concentrations. In the NanoString expression analysis, Mann–Whitney U test was used for the comparison between healthy and COPD subjects, and the Wilcoxon signed-rank test was used to compare 0% and 30% CSE. A *p*-value < 0.05 was considered significant.

## 5. Conclusions

In conclusion, there is a difference in the ER stress response to CSE in lung fibroblasts from healthy and COPD subjects. This difference indicates that COPD lung fibroblasts respond to CSE through apoptotic pathways. Deficiencies in regulation of ER stress-related apoptosis could be a potential reason for the progression of COPD. Although increases in some mediators of ER stress have previously been linked to COPD in some cell types, its response to CSE in lung fibroblasts tends to be atypical and therefore may be difficult to assess or target with therapeutic inhibitors developed in healthy samples. The atypical response in COPD subjects might also make future studies on COPD more difficult and should be considered when designing studies that investigate COPD.

## Figures and Tables

**Table 1:** Characteristics of healthy and COPD subjects from which BAL cells and lung fibroblasts were collected.

	BAL Cells		Lung Fibroblasts	
	Healthy n = 35	COPD n = 7	Healthy n = 24	COPD n = 19
Age, years ^a^	57 (20–73)	58 (57–68)	66 (20–74)	68 (52–77)
Male/Female, n (%)	13/22 (37%/63%)	3/4 (43%/57%)	8/16 (33%/67%)	11/8 (58%/42%)
Smoking status:				
Never-smokers, n	20	0	7	0
Ex-smokers, n	2	1	12	14
Current-smokers, n	13	6	5	5
GOLD stage 1/2/3/4, n	-	2/4/1/0	-	2/13/3/1
Packyears ^a^	0 (0–56)	43 (12–50)	1 (0–70)	43 (12–100)

Data are given as number of individuals (and %) or as ^a^ median (min-max).

## Data Availability

The data presented in this study are available on request from the corresponding author due to ethical reasons.

## Supplementary Materials

The following supporting information can be downloaded at: https://www.mdpi.com/article/10.3390/ijms25126600/s1.
