# Supplementary material for: Expression of Stress-Induced Genes in Bronchoalveolar Lavage Cells and Lung Fibroblasts from Healthy and COPD Subjects
Source: Int J Mol Sci. 2024 Jun 15;25(12):6600. doi: 10.3390/ijms25126600 (PMC11203587; doi:10.3390/ijms25126600)
Supplement: Supplementary file 1 [file ijms-25-06600-s001.zip › ijms-3033460-supplementary.pdf]

SUPPLEMENT

Expression of stress-induced genes in bronchoalveolar lavage cells and lung fibroblasts from healthy and COPD subjects

Martin Ryde[1], Nicole M D van der Burg[1], Frida Berlin[2], Gunilla Westergren-Thorsson[3], Leif Bjermer[1], Jaro Ankerst[1], Anna-Karin Larsson-Callerfelt[3], Cecilia K Andersson[2], Ellen Tufvesson[1]

[1] Respiratory Medicine, Allergology and Palliative Medicine, Lund University, [2] Respiratory Cell Biology, Lund University, [3] Lung Biology, Lund University

Supplemental Table S1. Characteristics of healthy and COPD subjects from which lung fibroblasts were stimulated with CSE.

	Healthy<br>n=9	COPD<br>n=7
Age, years[a]	66 (20-74)	72 (15-77)
Male/Female, n (%)	4/5 (44%/56%)	4/3 (57%/43%)
Smoking status:<br>Ex-smokers, n<br>Current-smokers, n	 6<br>3	 6<br>1
GOLD stage 1/2/3/4, n	-	1/4/1/1
Packyears[a]	20 (5-70)	43 (12-55)

Data is given as number of individuals (and %) or as [a]median (min-max)

Supplemental Table S2. Sequence of the primers used for RT-qPCR.

**Target**	**Forward 5' → 3'**	**Reverse** 5' → 3'
**ATF6**	GCT CTC TTT GCT GAA CTC GG	TGA GGA GGC TGG AGA AAG TG
**IRE1**	AGA TGC ACC AAG TAC AGC CT	CCT AAT GCC ACA CCT CAT GC
**PERK**	GAC ATG CTC TCT CCA TCC CC	AGG GCT ATG GGA GTT GTT GG
**CHOP**	GAT TCC AGT CAG AGC TCC CT	GCC TCT ACT TCC CTG GTC AG
**PSMB6**	GAA TCA TCA TCG CAG GCT GG	TGC AGA CAC TCT TCC TTG GT
**PSMA1**	AGG GCA GGA TTC ATC AAA TTG A	AAG CCC CGC AAT TGA GAT AC
**PSMD11**	TGG GGT GTG GTT TCT CTC TC	CCT CGC TGT GTA ATC GTG TC
**OXR1**	AGT GTT GAT TGC CAG GTT GC	GAC AAG GAA ATG ATG CCC TGT
**Bcl2**	TGC TGA AGA TTG ATG GGA TCG	TCA CGC GGA ACA CTT GAT TC
**NRF2**	CAC TTG TTC CTG ATA TTC CCG G	ATA GCT CCT CCC AAA CTT GCT
**GAPDH (Housekeeper)**	GAA GGT GAA GGT CGG AGT CA	TGG AAG ATG GTG ATG GGA TT

Supplemental table S3. Primary antibodies that were used for immunofluorescence staining and western blot.

Antibody target	Dilution in immunofluorescence	Dilution in western blot	Species	Article nr	Supplier
ATF6	1:250	1:500	Rabbit	Ab203119	Abcam
PERK	1:250	1:500	Rabbit	Ab65142	Abcam
IRE1	1:250	1:500	Rabbit	Ab37073	Abcam
CHOP	1:250	1:500	Rabbit	JM1031	Novus biologicals
PDI	1:250	-	Mouse	MA3019	Life technologies
GAPDH	-	1:1000	Mouse	Sc-47724	Santa Cruz biotech

Supplemental table S4. Proportions of the separate BAL cell types in healthy and COPD subjects

	BAL Cells	
	Healthy n=35	COPD n=7
Macrophages	83.2 (80.7-90.7)	86.1 (74.3-92.3)
Neutrophils	1.5 (0.5-3.9)	1.6 (0.2-10.5)
Eosinophils	0.5 (0.2-0.8)	1.1 (0.4-1.8)
Lymphocytes	7.5 (3.0-15.4)	4.1 (0.9-13.4)

Data is given as proportion (%) of the total BAL cell fraction.

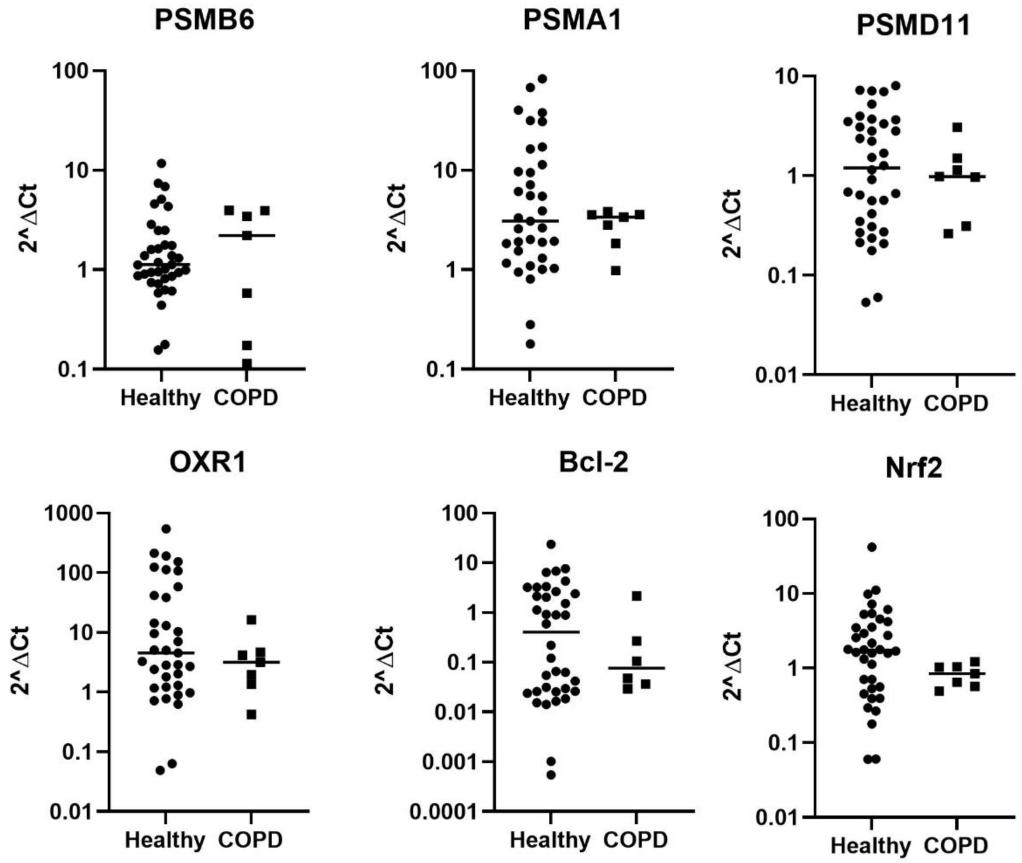

Figure S1. Gene expression of stress-related genes in BAL cells from healthy (n = 35) and COPD (n = 7) subjects. Mann-Whitney U test was used for statistical analysis.

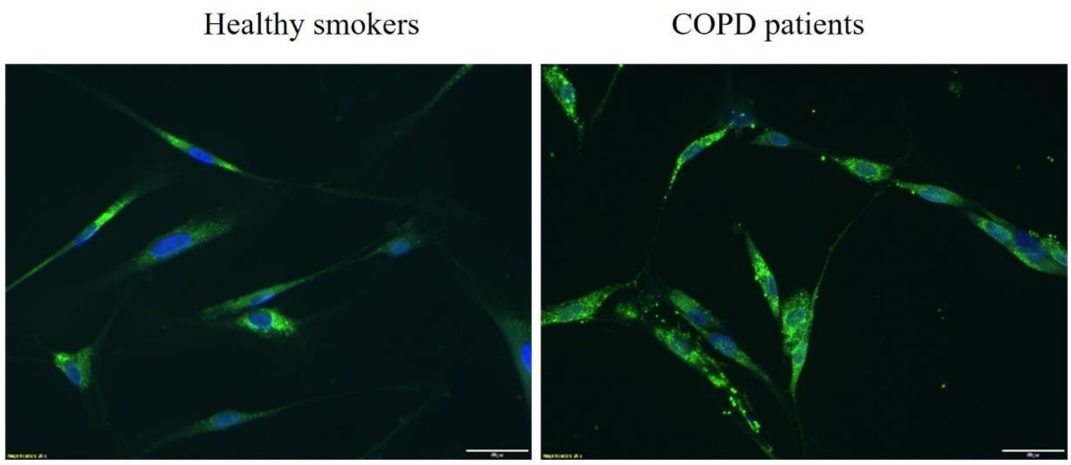

Figure S2. Representative images of lung fibroblasts from healthy smokers (n=4) and COPD patients (n=4). Green represents the endoplasmic reticulum (PDI) and blue represents the nuclei (DAPI).

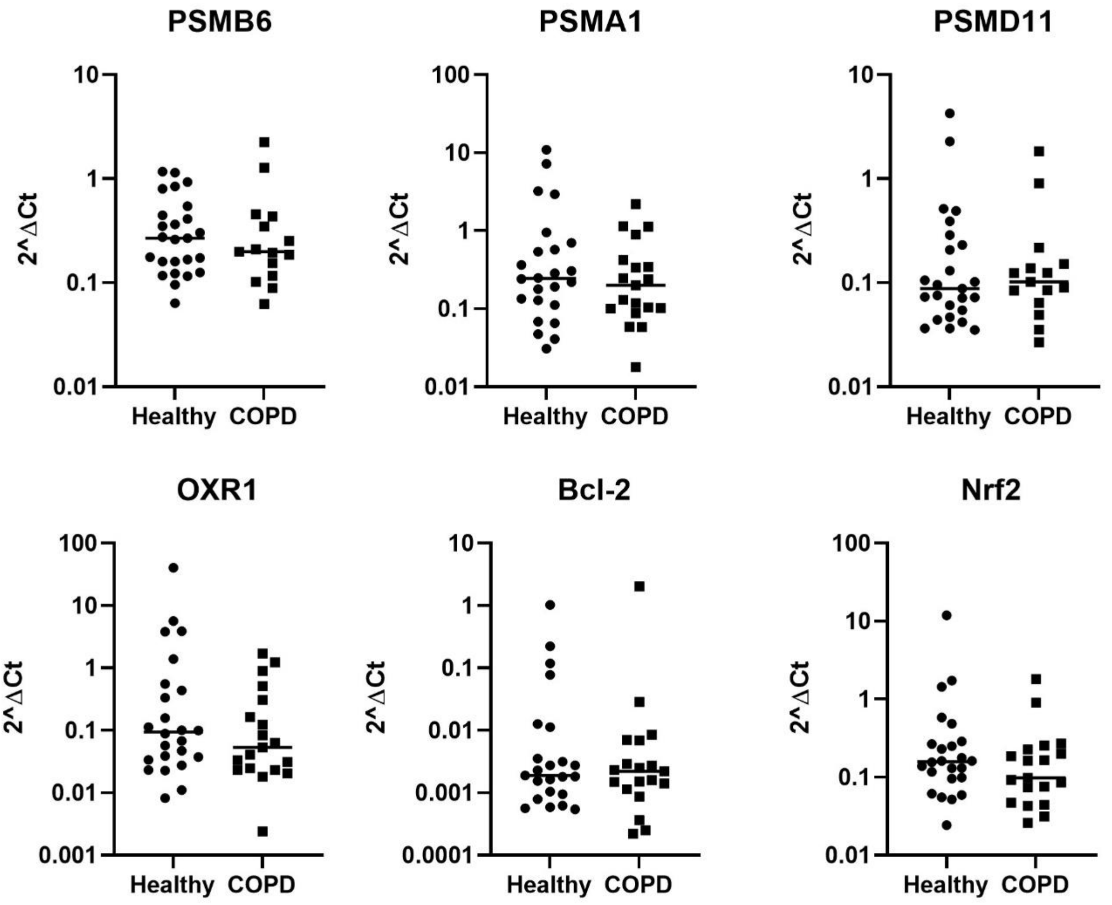

Figure S3. Baseline expression of stress-related genes in lung fibroblasts from healthy and COPD subjects. In PSMB6, and PSMD11: healthy n=25 and COPD n=16. In Bcl-2, Nrf2, PSMA1, and OXR1: healthy n=27 and COPD n=22. Data is presented as individual values and median. Mann-Whitney U test was used for statistical analysis.

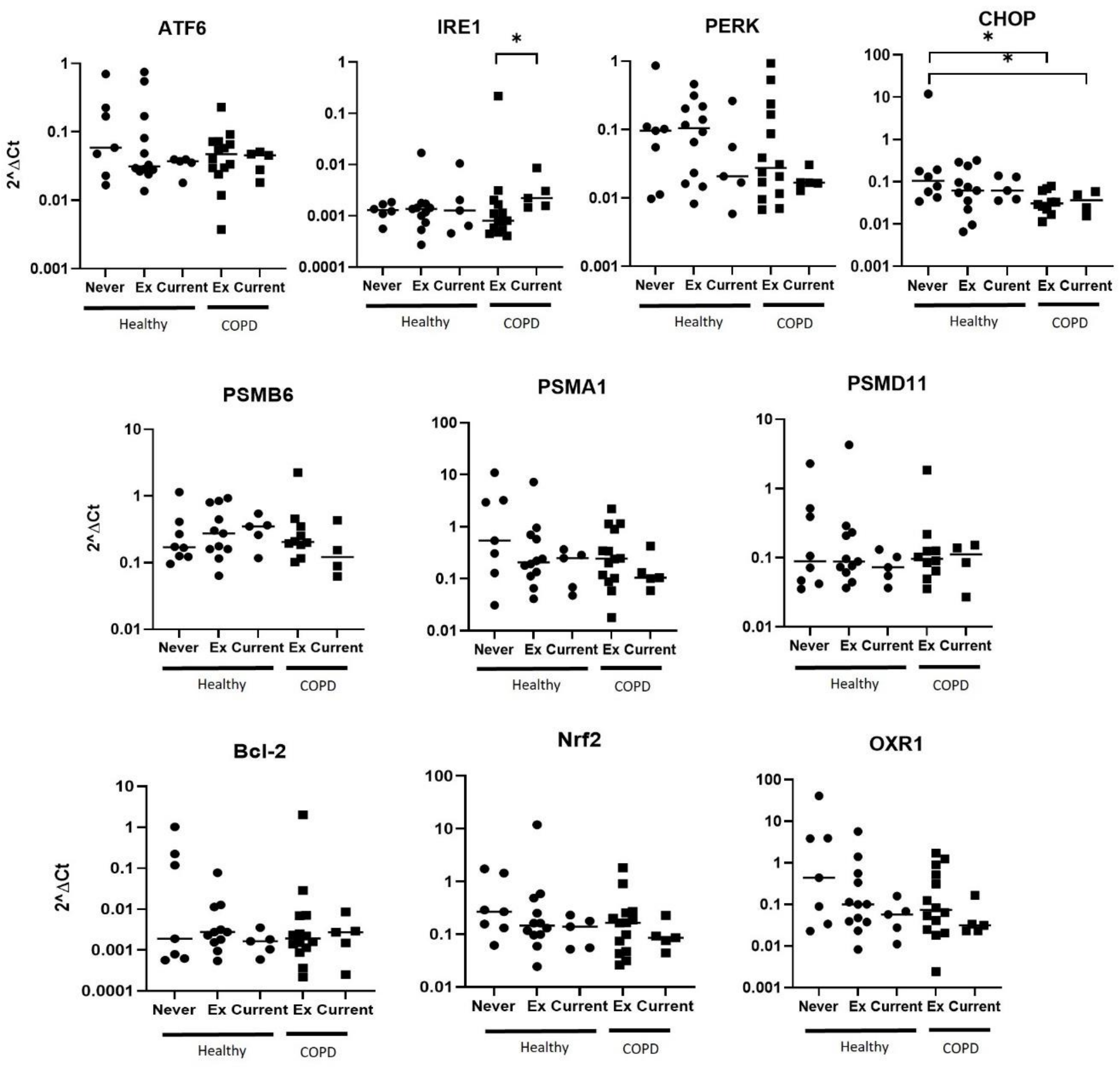

Figure S4 Baseline gene expression of stress-related genes in lung fibroblasts from healthy never-smokers (n=7), healthy ex-smokers (n=12), healthy current-smokers (n=5), COPD ex-smokers (n=14), and COPD current-smokers (n=5). Data is presented as median and IQR and Kruskal-Wallis test with uncorrected Dunn's test as post hoc was used for statistical analysis. * = $p < 0.05$.

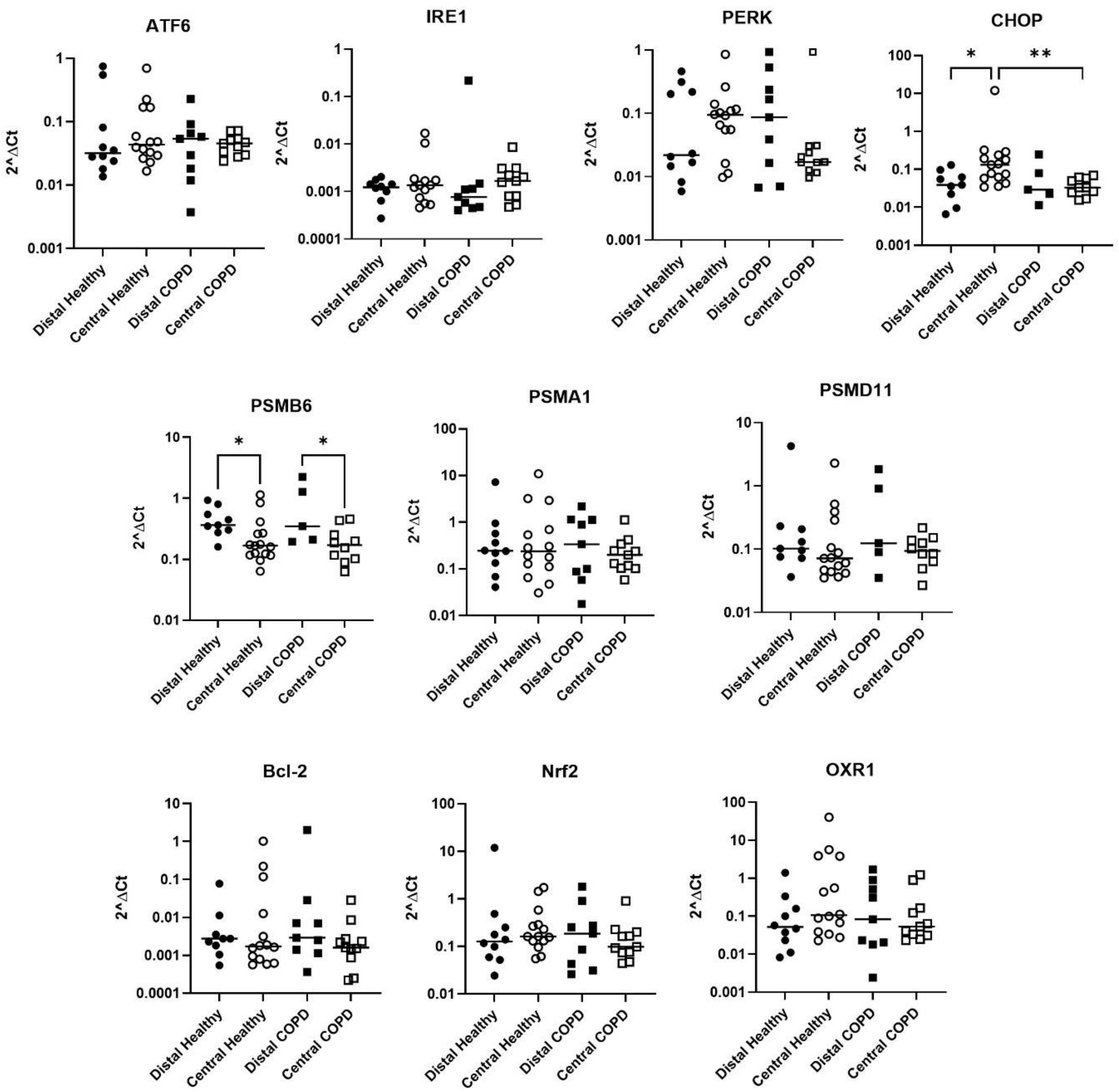

Figure S5. Baseline gene expression of stress-related genes grouped into distal and central fibroblasts in healthy (n= 9 resp n=15) vs COPD (n= 5 resp n= 10). Kruskal-Wallis test with uncorrected Dunn's test as post hoc was used for statistical analysis. * = p < 0.05, ** = p < 0.01

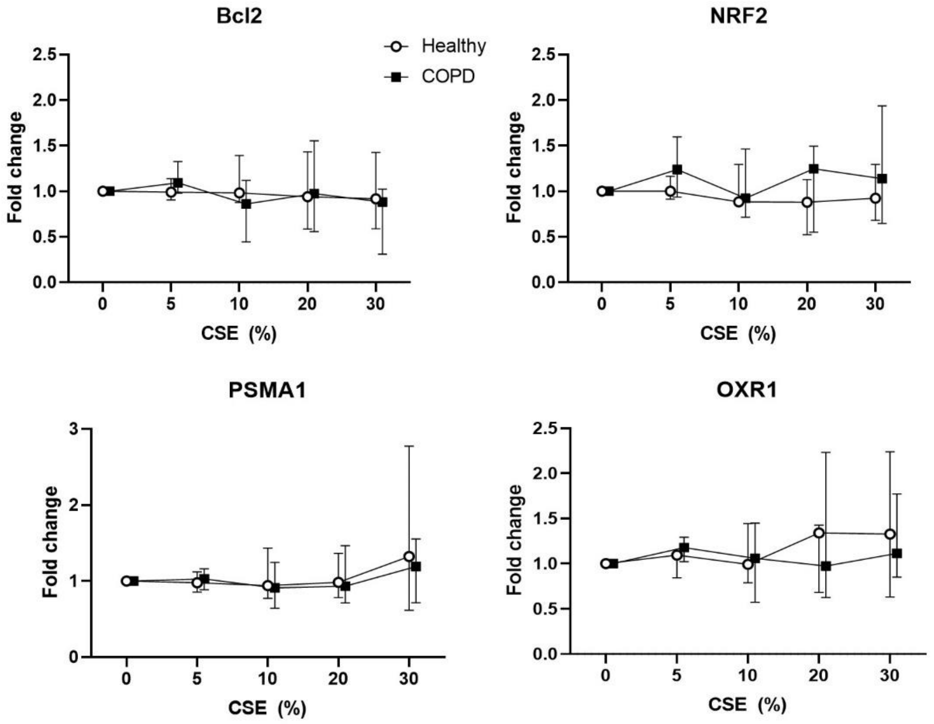

Figure S6. Expression of stress-related genes in lung fibroblasts from healthy (n=9) and COPD (n=7) subjects after stimulation with 0-30% CSE for 4 h. Data is presented as median and interquartile range. All values are presented as ratios relative to the respective individuals' result at 0% CSE. Mixed-effects analysis was used for statistical analysis.
